# Analysis of Dr. Jackson's Interesting Work on Idiopathic Fever; or "An Outline of the History and Cure of Fever, Endemic and Contagious, &c."

**Published:** 1799-07

**Authors:** 


*Analysis of* Dr. Jackson's *interesting work on Idiopathic Fever; or "An Outline of the History and Cure of Fever, Endemic and Contagious, &c."*

[Concluded from our third Number, pp 225——230.]

Chap. III.—*On the remote Causes of Fever.*

"IT is perhaps no longer disputed, that those derangements of the human frame, denominated fevers, whether *endemic* or *contagious*, owe their origin to two* sources only, viz. one natural and generally diffused over the surface of the globe, the other artificial and insulated: it is difficult, in many cases to define their limits precisely, but the matter is of such importance as to demand an attempt.

" The *first*, a vegeto-animal source, usually called *marsh miasma*, occupies the wide-extended bosom of the earth; it is generally diffused in the atmosphere, abounds more in some situations than in others, and is rendered more or less active by a great variety of causes,—causes, sometimes of regular connexion in the system of the world, sometimes apparently of accidental occurrence,—at one time obvious to the senses, at another obscurely perceived, or altogether imperceptible. This cause produces disease, and frequently such a derangement of the system as is incompatible with life; but *it is lost in its first operation.*

" The *second*, derived from an animal source, more expressly from an altered condition of the living human body, is confined in its sphere of action, communicated only by contact, by near approach, or by a medium connected with

---

* Dr. Robertson says, There are *four* great and dreadful sources of febrile infection, viz. marsh miasmata, jails, hospitals, and ships; and I avow that the infection of the three last sources is *one and the same*, consequently that the fever is the same; that fever from these last sources differs in no essential respect from fever arising from the *other* grand source of febrile infection; and, as the same mode of treatment is equally successful in all of them, I am led to conclude, *that febrile infection is the same throughout the universe*; and that the cure depends upon one invariable philosophical principle. See p. 8, 59, 188, &c.

with the source. This cause produces disease, and under that disease such a derangement of the system arises, as begets a multiplication of the original cause, which, extending itself to a certain distance from the source, propagates similar disease through a series of subjects.

"The *first* source affords the cause of the numerous forms and degrees of endemic fevers. These are observed to prevail in certain climates, in certain seasons, and in certain local situations of the same climate more than in others; they are upon the whole more frequent in tropical and warm countries, than in high latitudes and frozen regions; they are more frequent in spring, and particularly in autumn, than during the other periods of the year; and they are uniformly more frequent on sea-coasts and plains, near swamps and rivers, than in the interior and mountainous parts of a country.

"But, besides that these endemics are generally periodical, and in some manner connected with the causes which influence the *vegetation of plants*, they sometimes also appear irregularly and epidemically;—they affect strangers more generally and more violently than the natives of the district; and men under constant and active labour, less than those in a state of rest, or after desultory exertions.

"The source of this cause is known, and many of the laws which influence its action are clearly ascertained, but little or no progress has, as yet, been made in the discovery of its intimate nature and properties.—In a given time after exposure to the known source of the disease, more frequently at the expiration of a fortnight than any other period, the healthy action of the human body becomes suspended, impeded, or disturbed in different degrees; action of a new form arises, generally or locally exerted according to circumstances, which continues for a certain and stated time: it however often happens that the immediate action of the cause, or the effects consequent to that action, speedily and irrecoverably derange the organization of the frame, so that death ensues.

"Endemic fever is almost uniformly prevalent where vegetation is luxuriant, at least, where the requisites of a luxuriant vegetation greatly abound. In this manner, as vegetation is more luxuriant in tropical and warm climates, than in temperate and frozen regions, so notoriously is endemic fever." p. 105.

This argument in the following pages is placed in a variety of light, by Dr Jackson, and urged with a perspicuity and clearness of reasoning, we will not say peculiar to our author, but we may say, uncommonly gratifying to a medical reader; though perhaps the subject is not so important in this climate as that of the causes of contagious fevers. He concludes his remarks on this *first* source of the causes of idiopathic fever, with the following short recapitulation.

"It has been obſerved that this cauſe is loſt or changed in its firſt operation. It often rapidly deſtroys life; but it begets no proceſs in the human ſyſtem by which it can propagate itſelf. In ſhort, endemic fever may be, and often is, *epidemic;* but it is not *contagious.*—The ſource of endemic fever is a *natural* one, common to the whole earth; the ſource of contagious fever is *artificial*, produced by arrangements which take place among men in certain ſtates of ſociety.

On the cauſes of contagious fever, Dr. Jackſon, p. 109, ſays,—

"The cauſe, and conſequently the diſeaſe, is found in large, particularly in manufacturing cities, more eſpecially among claſſes of men confined to ſedentary employments, ſlothful and diſſipated in habits, oppreſſed with poverty, clothed in rags, confined and crouded in their appartments, and ſuffering from want of fuel. It is common in jails where men are crouded together, deprived of the benefit of pure air, ſuffering hardſhips of body, with various anxieties and afflictions of mind.

"It is, on the ſame account, frequent in work-houſes or poor-houſes. It ſometimes originates in, but is often tranſplanted to hoſpitals, where it ſpreads with rapid growth. It appears on ſome, though on rare occaſions, in the country among the families of poor cottagers. It is not peculiar to ſeaſon or to climate, but it appears oftener in winter than in ſummer, and in temperate than in hot climates; yet, as it depends every where upon adventitious and artificial cauſes, it ſometimes commits ravages in ſummer, and *it has even appeared in the torrid zone.*

"Such are the ſources from which this diſeaſe originates, and the ſituations in which it is moſt frequently found. Artificial conſtraint, and confinement in a narrow ſpace, by inducing a *new proceſs of ſecretion* in the living ſyſtem, ſeem to be the leading inſtruments in generating the cauſe. The organization of the human body is proved to be ſuch, that it does not preſerve a healthy action, unleſs under pure and free air; nor does it poſſeſs vigour, unleſs under *frequent changes of place, and active exerciſes,* calling forth the exertion of the moving powers. It indeed appears, that without theſe eſſential requiſites, the actions which ſupport life are not only languid, but they become diſeaſed, or fall into unnatural movements; in conſequence of which, the ordinary ſecretions are ſo changed, that though the actual exiſtence of fever be not apparent, ſomething noxious ſeems to eſcape from the ſyſtem, which, to a certain extent from its ſource, affects the health of others. In this manner it has often been obſerved, that perſons from jails, work-houſes, and other places of artificial confinement, though not at the time, and what is ſtill more remarkable, though not obſerved at any period to have laboured under formal diſeaſe, carry in themſelves or in their clothes, cauſes causes which occasion fever, in its most formidable aspect, to those who approach near to them. It is further to be observed, that the cause thus generated, speedily produces a fever in the body of a healthy man; and that the fever so produced is accompanied with such alterations in the secretions of the system, as to generate a cause, occasioning a similar disease, through an endless variety of subjects. This is a curious and an important fact. The fever which owes its origin to certain connexions with vegetable and *dead* animal matter, shows no disposition to propagate itself; the fever which arises from a connexion with the living human body, in a diseased state, multiplies with great activity; frequent ablutions, change of place, and change of clothing, under a dry, warm, and freely-circulating air, dissipate the cause, but do not change the nature of the disease. The cause is condensed, or rendered more powerful by states of the air, connected with cold and moisture: it is dissipated and weakened by the opposite. It is not extinguished by intense degrees of cold; but if adhering to the walls of apartments, or lodged upon clothes, it requires heat and moisture to call it into a state of activity. It is more powerful in its condensed, than in its recent and diffused state.

CHAP. IV. §. 1. *Cases of Contagious Fever*,—§. 2. *Cases of Endemic Fever.*

IN this chapter, Dr. JACKSON gives about twenty cases of each kind of fever, as some of the vouchers for the general doctrines and inferences contained in the work. The custom of giving a few cases on occasions similar to the present, is so common, that we presume Dr. Jackson thought himself bound to comply with it; but we think it seldom tends to promote the cause of science. It is almost impossible for any observer to detail cases with a sufficient degree of minuteness, for others to frame general principles from them so correctly as the observer himself. And since we consider Dr. Jackson's general principles and conclusions, as the most valuable parts of his work, we shall pass over these cases with only observing that, for the purpose of enabling readers to deduce general doctrines, they are not related with a sufficient detail of the doses of medicine, state of the heat, pulse, or tongue.

At p. 147 in this chapter, some appearances on dissection are given, of patients who died in consequence of the endemic fever, but these point no further than to conjecture on the cause of death.

CHAP. V. *Symptoms of Fever.*

"THE general character of that class of diseases denominated *febrile*, is not, perhaps, so comprehensively and so explicitly defined in the writings of authors, as to include and express every condition of febrile action.—

The horror, rigour, and accelerated pulse of authors, are only accidental circumstances; but an impeded or suspended—an irritated or irregular action—a changed condition of the state of the moving fibre, arising suddenly and acting generally, marked by its effects upon the functions of health and vigour, seems to constitute the primary and constant feature of the operation of a febrile cause*.

"The cause of endemic and contagious fever is radically different, but the mode of action, on the human body, is in many respects similar. In describing the gradations and forms of this action, it will be convenient and useful to class the appearances, according to certain general and prominent features, usually combined together, and seeming to depend upon certain conditions, or primary modes of operation. The deviations from health, arising from the action of the cause of fever, are different in kind and degree, and are differently combined, but upon the whole, the principal modes may be referred to the action of the moving fibre, diminished in force and energy, suspended or impeded in the usual range of freedom, disturbed in time, more frequent or more slow; or to a mode of action irritated beyond the usual degree.

"These modes, besides being different in kind and degree, are also such as to deserve the name of general at one time, the name of partial at another; sometimes they appear generally in the whole body, though different in degree; sometimes more particularly in one system or series of parts, sometimes chiefly in one organ, or the functions of one organ, and at other times in parts of an organ only, and partially in its functions."—p. 162.

SECT. I.

Dr. Jackson then proceeds to enumerate the symptoms which occur in each variety of febrile action, and begins with the *contagious* fever, the symp-

---

* Dr. Robertson states the general character of idiopathic fever in these terms: "Whenever men on board of a ship, or in a regiment, or any society or family, complain of being seized with chilliness, or alternate chills and heats, head-achs, sickness at stomach, universal pains, or as the sick express themselves, *pains all over them; or pains in all their bones, or joints; especially in their loins and back, with less or more debility;* and if their countenance is at the same time obviously diseased, whatever the other symptoms accompanying these are, I can, from experience, assure the reader, that a most virulent infection is present." p. 59.

On the causes of fever, Dr. Robertson says, "Whatever has a tendency to debilitate the system, may either be a remote or a proximate cause of fever, according to the constitution of the patients. A sufficient reason may be assigned for many people being seized with fever at the same time; which is, their being exposed to the same debilitating powers of heat, cold, drought, or wet; or sudden changes of these." p. 88.

ſymptoms of which he thinks may be naturally divided into *three claſſes*, viz.

Class I.—In which *ſuſpended or impaired action* is more conſpicuous.

This claſs admits of two varieties, depending on degree.—1. Slight in degree.—2. Aggravated in degree.

Class II.—In which *irritated motions* predominate.

Class III.—In which *particular organs or functions* are deranged.

The ſymptoms in the *firſt claſs*, when *ſlight in degree*, are

"*1ſt. Slight in degree.* The commencement of this form is uſually marked by a ſenſe of diſagreeable feeling at ſtomach, nauſea, vitiation of taſte, coldneſs of long continuance, an increaſed ſenſibility to cool air, or want of warmth, rather than horror or chillineſs, dimneſs of ſight, giddineſs, dulneſs of perception, languor, pains and aching of the joints, impaired and feeble action of the limbs, a ſad, deſponding, and lifeleſs aſpect of eye and countenance; the pulſe is ſmall, ſometimes, when ſuperficially attended to, ſeemingly not much changed from its natural ſtate, carefully examined, appearing to be feeble, languid, and confined,---ſometimes, but not always, more frequent than natural, ſometimes, but rarely, more ſlow: the degree of heat is moderate; judged by the ſuperficial touch of the ſurface and extremities, it does not ſeem to be above the natural ſtandard---upon the hollow parts of the trunk, or on preſſure, it appears to be pungent, though not ſtrong: the ſtate of the ſkin is uſually dry, ſometimes clammy and greaſy; ſecretions are impaired, with great deficiency of alacrity in the functions.

"The above foundations of derangement being apparent in the firſt twenty-four hours of the diſeaſe, the greater number of ſymptoms increaſe in degree, or ſuffer ſome change of form during the continuance of the courſe: the appearances of the tongue are irregular---ſometimes the tongue is moiſt and little changed; ſometimes it is rough and moiſt, or with a thin coat, firmly adhering, ſometimes white, ſlimy, and foul; it is ſeldom dry in this ſtage of the diſeaſe, but it is not unfrequently covered with a ropy ſaliva, ſmooth, and without papillæ. Some degree of nauſea is often preſent, but there is ſeldom any vomiting. The body is ſometimes open, ſometimes bound; when open, the evacuations are, for the moſt part, ſmall and ineffective: there is uſually an unpleaſant taſte in the mouth, or want of taſte; almoſt always a dry or a greaſy ſkin, a ſullen aſpect, a pulſe not much changed in time, but feeble and unenergetic in the ſtroke; pain of the limbs and joints, like the achings in the cold ſtage of intermittent, ſometimes tenderneſs of the touch; bad reſt---dreams and wanderings often diſturb the ſleep. At a certain period, ſometimes on the third, oftener on the fifth, and ſtill oftener on the ſeventh day, theſe ſymptoms undergo a great and material change; the action of the ſyſtem, particularly the action of the arterial ſyſtem, developes, ſecretions are reſtored, and and the figns of health return: or inftead of health, a new train of morbid action commences. The caufe, which had hitherto only in a flight degree impaired the energy of the vital functions, either by a fudden acceffion of power, or by an increafe of aptitude in the fubject, acts with vigour, and fometimes rapidly overwhelms life. Where the action is general in the fyftem, the progrefs is ufually more gradual, and the term of exiftence is longer protracted; where the action is partial, the functions of parts become oppreffed, and death takes place fpeedily.

"The figns of gradual extinction are chiefly found in a pulfe, fmall, weak, fometimes frequent, and always feeble, wrapped up as it were in itfelf, retiring from the furface and extremities of the body, irregular, intermitting, and finally failing; in a correfponding decline and failure of animal heat, in a ftate of fkin dry and impervious, fometimes greafy, clammy and dirty, fometimes purplifh or livid; in fecretions impaired or fufpended, in drowfy torpor, but diminifhed fleep, with an imperfect poffeffion of intellect. The tongue during this progrefs, particularly where the progrefs is not rapid, is often black and dry, covered with a footy pellicle, fometimes fmooth, clear, and gloffy, the lips too are generally dry, and a black cruft or pellicle fometimes covers the teeth and gums. Where the action is partial, the appearances are more irregular:—irritated motions are varioufly combined with fufpended or diminifhed energies, and, according to circumftances, the fymptoms are of great variety, and in fome refpects oppofite in their natures. In one cafe, the head is principally affected:—delirium, different in degree, and different in kind, occupies the chief notice; in another, affection of the moving powers gives rife to a multitude of alarming fymptoms, ftartings, tremors, convulfions, &c.; the cheft fuffers principally in one, fo that the functions of the lungs become fuffocated and oppreffed; the ftomach and alimentary canal have a great fhare of the fufferings in all and at all times; in the latter ftages, thefe organs often feem to lofe power in a more efpecial manner—the abdomen becomes inflated, involuntary purging takes place, accompanied, on many occafions, with involuntary or unconfcious difcharges of urine.

"It likewife happens frequently at this period of change, that, inftead of ceffation of difeafe, a new train of morbid appearances arife, which are different in afpect from the preceding, and lead for the moft part to different iffue; they are chiefly confpicuous in the changed action of the vafcular fyftem; the pulfe, from fmall, weak, and confined, deep, and as it were funk in the arm, becomes gradually open and full; the whole fyftem feems to fill and expand, or the tide of circulation flows freely to the furface and extremities of the body, the mind alfo becomes cheerful, and even gay, delirium of the gentle, but lively fort, is a frequent occurrence, and often appears to be a leading feature; fleep is found, or difturbed only by pleafant fancies and wanderings, the energy of the functions is reftored, the fecretions refume their natural courfe, and marks of crifis are generally decided and final.

final. During this gradual progrefs to health, the tongue is ufually dry; a coat or covering forms upon it, becomes thick, and feparates at the critical period, moft ufually the fifth or feventh day from the new train of action.

" 2d. *Aggravated in degree.* Appearances, in the more aggravated degrees of this form of febrile action, vary according to the feries of parts principally affected---the different fhades are painted in the countenance. The figns of the commencement are not very different from thofe of other fevers, viz. difagreeable fenfations at ftomach, naufea, flatulence, liftlefsnefs, languor, feeble action of the moving powers, fenfe of cold, or want of natural warmth, head-ach, from heavinefs and confufion, or mazinefs (as the foldier ufually terms it) to a degree, which gives the impreffion of being knocked down by the blow of a hammer---a ftupor like that of deep intoxication---a total inability of holding up the head. The appearance of the eye and countenance indicate ftrongly a material deviation from health, but an accurate picture is not eafily conveyed in words: the eye is fometimes gloffy; the look vacant and ideot-like, torpid, fad, and defponding; the countenance collapfed, dry, and withered, like a plant nipped by froft, or failing from want of rain,---fometimes flaccid and dirty, as if wafhed in greafy water ---fometimes full, fwollen, torpid, and inanimate, like a ftatue---fometimes lurid, dark, and grim, refembling a piece of mahogany; this laft is often connected with heavy breathing, deep fighing, and ftricture of the cheft.

" The fymptoms above-mentioned appear in the firft twenty-four hours, and increafe in degree, according to their feveral forms, for different fpaces of time---three, five, or feven days. The countenance, during this period, becomes daily more flaccid, withered, and dry, more dirty and greafy, more fwollen and inanimate, more lurid and dark; in all there is a dufky hue, with a lighter or deeper fhade of yellow; the changes of the eye obferve the fame rule of progrefs---the veins become large, and the motions are languid, with confiderable changes of colour; the pain of the head, if fevere in the commencement, is often changed at a certain period, to a fenfe of mazinefs or confufion, a want of power to command thought. There are fometimes mutterings and wanderings during the night, with want of fleep; but delirium (properly fo called) is not common in this ftage; where the courfe is rapid, it fometimes precedes convulfion; it is often the fign of change of action, and marks the commencement of a developement of the energies of the vafcular fyftem. The pulfe in this form of fever does not offer much information to fuperficial obfervations; it is many times little altered from what it is in perfect health, fometimes neither more frequent nor more quick, but generally, when minutely attended to, lefs expanfile, without force of contraction, or free dilatation; a want of energy of ftroke, a defect of irritability, feems to characterife it, when clofely examined---curforily noticed, it often feems neither fmall nor weak. This nearly natural ftate of pulfe continues, with more or lefs variation, for fome time; at a certain period, it becomes frequent quent, quick and active, free and expanfile; or the torpor increafing, it wraps itfelf up, withdraws from the furface and extremities, and finally fails. The ftate of animal heat, another of the figns from which an opinion is ufually formed of the nature and degrees of fever, affords little remark to common notice. In touching the arm lightly, the heat feldom feems to be increafed—in preffing it clofely, it is often found to be cauftic and pungent, unpleafant, and differing from the nature of warmth; it is deep-feated, irregularly diffufed, concentrated about the præcordia, deficient in the extremities. The fkin, correfponding with the afpect of the countenance, is fometimes dry and impervious, flaccid and withered, fometimes dirty, greafy, and clammy, fometimes livid, and in a manner marbled about the tendinous parts, the knees, feet, and hands, befpotted with petechiæ on fome occafions, and on many fore, or tender to the touch: gangrenous fpots are not uncommon on the feet, hands, knees, nofe, and ears; extenfive and large mortifications fometimes make their appearance on other parts; pains or achings, like thofe in the cold ftage of intermittents, are common and diftreffing. Some idea of the ftate and progrefs of fever may be, and ufually is, formed from the afpect and condition of the tongue. In feveral inftances, the tongue does not appear to be materially changed, being moift, and with only a thin covering of mucus; in others, it is moift, fmooth, and without the ordinary prominence of papillæ,—it is fometimes rough, but cannot be faid to be foul, at other times it is covered with a mealy, milk-white pafte, of different degrees of thicknefs, the mouth overflowing, at the fame time, with a ropy faliva:—fimilar alfo to the afpect of the countenance, the tongue is fometimes torpid in its motions, cold and pale, or large, fwollen and livid, or lead-coloured. In the progrefs of the difeafe, the coat, by which the tongue is covered, ufually becomes thicker, fometimes dry, rough, brown, and even black: about the period of crifis, termination, or change, this covering loofens and feparates at the edges, and at laft through the whole extent; the tongue thus becomes clean; but if the difeafe runs on through another period, it turns rough, dry, often black, covered with a black cruft or pellicle extending fometimes to the teeth and gums; fometimes it remains clean, fmooth, gloffy, red and parched.—Thirft often bears a correfpondence to the ftate of the tongue and fauces; yet thirft is fometimes intenfe, without the correfponding appearance, or inconfiderable, where according to the ordinary rules it would be fuppofed to be great. The tafte is changed, or depraved; there is feldom a relifh for food, though food, at leaft fpoon-meat, is fometimes fwallowed with indifference; naufea is not uncommon, but vomiting is rare; yet vomiting fometimes happens in confequence of a change of determinations. The functions of the alimentary canal are much difturbed; in fome cafes there is coftivenefs, even refifting ftrong purgatives, in others there is purging, but the evacuations are feldom effective, they are watery or fmall; fevere gripings or pains are not uncommon: fullnefs, tenfion, and inflation of the hypochondria, and, towards the latter periods, involuntary ftools are among the ordinary appearances.

Secre-

Secretions are generally diminiſhed, and among theſe, the ſecretion of urine is ſcanty, with complaint of pain, and difficulty in rendering it. There is uſually a want of reſt in this form of diſeaſe, at leaſt a want of refreſhing ſleep, but anxiety, toſſing and change of poſture, are by no means common; there is however, on many occaſions, a ſenſe of ſtricture on the cheſt, a dry and teazing cough, often alternating with affection of the head.

"The foundations of the above derangements being laid in the commencement of the diſeaſe, the ſtructure advances to a given point, with different progreſs. Sometimes on the third, oftener on the fifth, and oftener ſtill on the ſeventh, the action of the ſyſtem becomes excited, the powers of life emerge, and criſis takes place; or fever being in ſome manner ſuſpended, ſufferings abate or ceaſe, recovery goes on for a few days, when diſeaſed action recurring ſuddenly, life is overwhelmed or brought into danger; or further, inſtead of termination or ſuſpenſion of morbid action, the cauſe ſeeming to receive an acceſſion of power, or a change of direction at the above periods, the vital energies become generally or locally oppreſſed, and death is the conſequence.

"Class II.—*Irritated Motions*. The primary action of the cauſe of fever is obſcure; but, at a certain and early period, action, irritated, irregular, and apparently increaſed, is ſo conſpicuous in the vaſcular ſyſtem, with unuſual commotion of the moving powers, as to characterize a form of fever, which deſerves particular conſideration from the multiplied variety of its appearances, and the numerous accidents which happen to life from its tumultuary diſturbances. The attack of this form of diſeaſe is uſually ſudden, the ſenſe of cold or even horror is conſiderable, and frequently alternates, during the firſt hours, with ſenſations of burning heat; the headach is often intenſely ſevere, particularly the pain of the forehead and temples,—it is frequently preceded by giddineſs, vertigo, and temporary loſs of ſight; the eye is often muddy, confuſed, and red, ſtaring and prominent, it ſometimes ſeems to blink, or ſhun the light; the countenance is fluſhed, or rather overcaſt and grim, often agitated and confuſed; the tongue is generally foul, covered with a mealy or milk-white paſte, when moved it is ſometimes tremulous; the thirſt is great; the ſenſations at ſtomach unpleaſant; nauſea is not unuſual, and even vomitting occurs ſometimes; pains in the legs and joints are ſevere; pains ſhooting along the legs, ſhoulders and arms in repeated exploſions, or with ſenſations of gnawing, and as it were, tearing, are ſometimes extremely diſtreſſing; a general ſoreneſs or tenderneſs of the touch is not uncommon, in all caſes, however, different in its nature from the tenderneſs or pain of rheumatiſms; the uneaſineſs and anxiety are frequently great; agitation, tremors, ſtartings, convulſive motions, ſtricture of the cheſt, irregular evacuations of the bowels, with gripings, ſevere pains and ſpaſms, are occaſional, but fluctuating ſymptoms; the ſkin is uſually hot and dry; when the body is preſſed cloſely, the heat often appears cauſtic and fiery, deep or concentrated; it is unequal in different parts of the body; the pulſe is uſually quick and frequent; irritation and motion are increaſed, but force force and energy are wanting; the usual freedom of the stroke is confined; secretions are irregular, and generally impaired.

"These symptoms, which appear in the first twenty-four hours of the disease, increase with some variety of progress to certain periods, at which time, changes or terminations, sometimes favorable, sometimes fatal, are observed to take place. The modes follow the ordinary rules in fever; in one case, the action of the vascular system becomes vigorous, the arterial pulsations expand and final termination is the consequence; in another, the irritated motions of the arteries abate, or cease, the course of the disease seems to be suspended, some portion of health returns; in some instances, imperceptibly established, in others, suddenly deranged, by a recurrence of morbid action; in a third, the irritated or increased action subsides rapidly, while the vital energies being exhausted generally, or organs locally destroyed, death ensues with more or less variety of appearance. The different events are, in some measure, connected with certain days in the following manner. In the more concentrated forms, the irritated action often subsides on the third day, sometimes fatally, sometimes, indeed, with hopes of returning health, but with still greater suspicions of sudden and dangerous recurrence. In forms of somewhat less violence, the changes of the fifth have similar issue. The seventh is the great critical day of regular fevers of moderate violence; the terminations are oftener final, or changes more distinctly marked than on the others; at this period the powers of life subside rapidly, emerge speedily, or a new train of action commences, in the progress of which the action of the vascular system is developed, and health is finally established.

"The appearances, in this fever of irritation, are, as they might be expected to be, very irregular; pains and spasms in different parts of the body are severe, the pulse is irritated, and sometimes excited to a high degree of action, the heat is ardent, caustic; sometimes making an impression like actual fire, the thirst is great, the tongue dry—sometimes rough, foul, and black; starting tremors, convulsive twitchings are frequent; the breathing is often oppressed, stricture and affection of the chest frequently alternate with delirium and affection of the head: vomiting sometimes occurs, prodigious in quantity and irrestrainable, accompanied with a small pulse and cold skin; on some occasions the same is the case with purging; the urine is suppressed, sometimes it is bloody; the functions of the liver are also suffocated in some instances, or there are appearances of deep jaundice; hæmorrhage from the nose, though not common, sometimes takes place, and gangrene or blacknesses, sometimes of considerable extent, appear on the extremities, or other parts of the body.

"Class III. *Local Fever.* The cause of fever generally affects the system extensively, but on some occasions the principal action seems to be excited upon distinct organs, or upon the functions of organs; the dysenteric and peripneumonic forms are the most common and the most formidable. In these forms, forms, pains, fpafms, and marks of irritation are fometimes confpicuous; fometimes, more particularly in the dyfenteric form, the action of the arterial fyftem is very little difturbed; the courfe of the peripneumonic form is ufually the most rapid, the dyfenteric fometimes continues feven days or a fortnight, without very materially impairing the functions of the general fyftem; but it is common to both, that the caufes, which change or reprefs the local difeafe, feldom fail to diforder the exifting economy of the frame: thus peripneumonic fever, or affection of the cheft, is often changed to delirium, or affection of the head; dyfentery changed, or fuddenly repreffed, is alfo followed by general fever, delirium, fpafm, and convulfion.

The above are the more common forms and appearances of the fever, which prevailed among the Britifh troops, in the different fervices on which the author was employed. It was remarked before, that the principal diftinction of fever confifts, in an affemblage or train of motions irritated to an unufual degree, or deficient in ordinary force and energy:—thefe are not however fo pure and fimple in all cafes as here defcribed; one character may be obferved to prevail upon the whole, but it is more or lefs mixed on different occafions;—the motions, for inftance, feem irritated in one part of the body, and torpid in another at the fame time, and the afpect of the general mode of action changes repeatedly during the continuance of the difeafe. In the commencement the motions are fometimes greatly irritated,—the fever, in common language, runs high; at a certain period this irritation fubfides, and a courfe of deficient energy or torpor enfues; on the contrary, torpor and deficient energy are fometimes confpicuous in the early ftage; at a certain period the motions become irritated, the action is increafed, the powers of the circulating fyftem expand, and the energies of life are reftored.

"It is not pretended to determine the caufes upon which the varieties of difeafed action in fevers depend, but it will not be without ufe to notice the circumftances, with which certain modes are primarily connected, or from which they feem accidentally to change. In encampments, in cool and wet weather, under deficiency of clothing, tedious and irkfome confinement, the *dyfenteric* is often the moft confpicuous form;—this often ceafes on removal to warm and dry lodging; genuine fever then arifes;—in crouded barracks, in crouded fhips, among fubjects depreffed in mind, inactive in body, the form of fever is ufually a form of *deficient energy*,—of longer and fhorter duration, and greater or lefs intenfity, according to a variety of accidents; in the moft concentrated fources of contagion, as in crouded hofpitals, the action of the caufe is ftrong, fometimes reprefenting, in its attack, a form of apoplexy, which, where the habit poffeffes little power of refiftance, rapidly overwhelm life. Thus upon thofe who live in confined air, and who do not exert themfelves in body or mind, the caufe of the difeafe feems to act by an operation of *depreffion or fufpenfion*, and death takes place,

 often often without much ftruggle or difturbance; in thofe, who, expofed to the fame caufe, go abroad into the open air, and, more particularly, who are active in body and mind, the febrile motions are *irritated, irregular*, frequently increafed in force, terminating in eftablifhed re-action, or deftroying the organization of parts by violence of effect. In crouded hofpitals, where dirt, naftinefs, and bad air prevail, the relapfes of the difeafe are frequent; the forms indicate deficient energy in the general fyftem, or local derangement from accidental weaknefs; the events are often unfortunate. Under a free ventilation of air, and the advantages of perfonal cleanlinefs, the action in relapfe is irregular; pain, fpafms, and purging, come fuddenly, and fuddenly ceafe; intermitting forms alfo occur often, but they do not obferve the fame regularity of period as is ufual with pure endemics."

SECT II. *The Symptoms of Endemic Fever.*

This admits of the following diftinctions, viz.

1. Into a form, where irritation, tumult, and excitement of the vafcular fyftem are chiefly confpicuous in the early period; local derangement and diforder of important functions in the latter.

2. Into a form of fever, where the action of the vafcular fyftem appears to be deficient or oppreffed, the moving powers impaired in their energies, or rendered irregular in their motions.

3. Into the fcorbutic, or low form: And 4. Into fevers of type.

I "The *firft* form occurs moft commonly in vigorous and athletic habits, and it often occurs, under the circumftances of preceding defultory exertion, or tranfgreffions of the rules of temperance. The invafion appears upon a general view to be, for the moft part, fudden or inftantaneous; but, upon accurate inquiry, languor, head-ach, or fome obfcure deviation from health, will ufually be found to have preceded the formal attack, by twelve hours fometimes by a longer fpace. Head-ach, if not actually the firft, is among the firft fymptoms of this, as of other fevers; and the nature of the head-ach is different, in different fubjects; fometimes it is almoft infupportable, confined more particularly to the forehead and temples, accompanied by fenfations of tightnefs over the eyes, turgefcence and ftarting; fometimes the pain is more generally diffufed, dull and obfcure; but it, for the moft part, differs from the head-ach of ordinary caufes, though the difference is not eafily defined. Head-ach is almoft always prefent in the commencement, in fome degree or other; it fometimes abates in the courfe of the firft twelve hours, frequently in the firft forty-eight, and almoft always before the termination of the third day. During the feverity of the pain, the forehead is fometimes hot and burning, fometimes

ſometimes cold and clammy. It happens alſo, and not unfrequently, that together with, or inſtead of head-ach, the attack is uſhered in by giddineſs, vertigo, drowſineſs, or ſtupor like deep intoxication: fits of apoplexy, hyſteria or tetanus, are obſerved ſometimes, but they are upon the whole rare, and by no means charaсterſtic of peculiarity. The ſtomach, one of the organs primarily and principally affected in the commencement of fever, is for the moſt part peculiarly affected in the early ſtage of this diſeaſe; vomiting is not common, but nauſea, with a taſte of copper in the mouth, is uſual, as are anxiety, flatulence and other diſtreſſes, not eaſily deſcribed, as not referable to diſtinct heads. The ſenſations of liſtleſſneſs, languor and averſion to motion common in the commencement of fevers, are preſent here, but have not any peculiar qualities. When the invaſion is ſudden, the debility or loſs of power is more complete. Alternate chills and fluſhings of heat are not unuſual; in ſome caſes they occur at intervals for the firſt twelve hours; the ſenſation is unpleaſant, but the cold is ſeldom ſtrong, or amounting to rigour; in others, chills are not perceivable, the ſenſation of heat prevailing from the beginning. Together with theſe, the eye and countenance are confuſed and agitated, the pulſe diſturbed, frequent, ſmall, heſitating, or ſeemingly oppreſſed under the immediate attack.

The following appearances are noticed during the firſt twelve hours, though with ſome difference of order and degree in different ſubjects. The eye and countenance expreſs ſome marks of peculiarity,—known to actual obſervers, difficultly conveyed in deſcription. The eye is ſometimes watery, ſad and deſponding, ſometimes agitated, red and diſturbed, as if ſuffering from the ſmoke of green wood; the pupil ſometimes appears to be preternaturally contracted, ſometimes preternaturally dilated, but its appearances cannot be ſuppoſed to afford a criterion or diſtinguiſhing mark of the diſeaſe; the eye-balls are often much agitated, ſtaring and protruded. The countenance is uſually, but not always, fluſhed; it is generally clouded, agitated, expreſſing a ſecret ſuffering of diſtreſs, not indicated by other external ſigns. The tongue is generally white, ſlimy, and moiſt, ſometimes apparently clean, or covered with ſo thin a covering of mucus, that the red ſurface below ſhines through it, forming a colour reſembling that of lead, ſometimes it is ſmooth, it is ſeldom dry and rough. Thirſt is irregular and uncertain; where nauſea prevails, it is ſeldom great, yet intenſe thirſt and nauſea ſometimes meet together. The pains in the joints, limbs and back are often ſevere and diſtreſſing; they reſemble pains in the cold ſtage of intermittents; the pains of the calves of the legs are ſometimes acute, the muſcles ſeeming to be in a certain ſtate of ſpaſmodic action. The pulſe is uſually frequent, ſmall, confined, concentrated or deep, as oppoſed to expanding and open. It is ſometimes times agitated, vermicular, confufed in an uncommon manner, almoft always it is much difturbed. The heat, during the firft twelve hours, feldom appears great, if the fkin be touched lightly; more clofely preffed, the fenfation of heat is cauftic and pungent. The fkin is ufually dry; if damp, it imparts the idea of a fpafm exifting on the furface, for perfpiration, with expanding pulfe and relief from fufferings, rarely takes place. If the fkin be moift, the moifture is clammy, as in agonies,—different from the warm and fluid moifture, which follows a relaxation of extreme veffels. To eftimate this properly is a matter of fome importance in forming an opinion of prognoftic. In thofe fweats, which terminate the paroxyfms of the remitting fevers of natives or feafoned men, the pulfations of the arteries become full, expanding, and, as it were, rife to the furface, the perfpiration is fluid, copious and general, with foftnefs of the fkin, and with the fenfation as if a load of weight and diftrefs were removed from the fyftem; fecretions, are, in fome meafure, reftored, and the countenance becomes to a certain degree cheerful and ferene. In the abatements which take place towards the clofe of the firft twelve or twenty-four hours of this difeafe, the appearances are often flattering and fometimes fo ambiguous, as to deceive an unexperienced practitioner; but the following marks will, in fome meafure, help to note diftinction; the fweat is feldom copious and general; it is, for the moft part, confined to the upper parts of the body; it is not ufually fluid and free, but has fomething of clamminefs joined with it; the pulfe does not expand; an impreffion of exifting fpafm or confinement,—of imperfect dilatation and feeble contraction of the artery, ftill remains. Yet in fome inftances, the changes are fo material, the relief fo evident, that it is only after much experience, that a perfon is brought to doubt of the prefence of remiffion; indeed in people accuftomed to climate, fuch abatements may fafely be efteemed remiffions and acted upon as fuch; in Europeans, newly arrived in warm latitudes, they require to be regarded in a favourable light with much caution.

"About the termination of the firft twelve hours, the tumult and agitation defcribed above in fome degree fubfide; the appearance of the eye, though not ferene, is lefs wild, and expreffes a fenfation of relief; the violence of the head-ach diminifhes, or the nature of the pain changes; the countenance brightens; the patient, even the phyfician, is often flattered with hopes of remiffion,—but they are fallacious hopes, and feldom of long duration. In the courfe of a very few hours, at fartheft, the fymptoms recur with aggravation, and with qualities in fome refpects differing from the preceding. The pulfe which, during the firft twelve hours, was ufually fmall, frequent, irregular, or confufed, becomes quick, hard, tenfe, more equal in time and force, but confined, or without a free dilatation, or energetic contraction of the artery. The heat of the body, particularly on the head and trunk, is burning, fiery, and concentrated; if the skin be touched lightly, it sometimes does not appear to be uncommonly great—pressed closely, it is often so intense, as to be indured with pain, communicating the sensation of actual fire, or of sharp instruments darting into the fingers. Thirst is irregular, much connected with the state of the tongue and stomach; where there is nausea, with a moist and foul tongue, it is seldom great, at least, if there be a desire of drink, there is also an aversion to the act of swallowing: where the mouth and tongue are dry, thirst is generally intense. The pain of the head, which had in some degree abated, recurs again, but it recurs with sensations differing from the former; marks of increased determination are now evident, the pulsation of the temporal, and particularly of the carotid arteries, is sometimes so violent, as to cause the head and neighbouring parts to shake; there is also a sense of fulness, weight, and heaviness through the whole head, sometimes with drowsiness and coma, but without the power of sleeping; the recollection is confused, and not under command, but that derangement of the reasoning faculty, properly called delirium, is a rare occurrence. The countenance is highly flushed, sometimes dark and cloudy; the eye is muddy and inflamed; the urine is scanty, sometimes suppressed: the bowels are torpid, difficultly moved by purgatives, or moved by starts,—the evacuations, watery and in excess, do not afford relief; the skin is generally dry, and the heat is unequal—great in the trunk, as formerly observed—diminished, or deep-seated on the extremities. Sighing, deep breathing, anxiety, and undescribable fidgetting, or desire of constantly changing posture, without complaint of pain, or specified object, are common attendants of this stage of the disease; and these undefinable uneasinesses may be regarded as the surest signs of the existence of concentrated or yellow fever."

Want of room obliges us to refer our readers to the work itself for the remainder of this enumeration, as well as many important particulars in the following forms:—

II. "The *second* form, as observed among Europeans newly arrived in the tropical regions, chiefly occurs among men who lead inactive and indolent lives, who are confined in the less pure air of crouded towns, crouded barracks, or crouded ships, who are under the impressions of ennui, chagrin, and fear, or who are constitutionally deficient in energetic exertions of mind and body. According to the natural qualities of the constitution, the accidental circumstances of the individual or modification of cause, the disease seeems to be at one time characterized by severe local pains, spasms, or tremors, by general and undefinable uneasiness, or fidgeting—at another time by torpor, by indifference of mind, and impaired sensibility of body.

It

It is ſometimes ſudden in its attack, ſometimes gradual in its approach.— When it aſſumes the diſtinct febrile form, the leading circumſtances of its hiſtory are the following:—

" It uſually commences with giddineſs, even to blindneſs, pain of the head, faintneſs, ſickneſs diſagreeable ſenſations at ſtomach, weight and oppreſſion, even nauſea and vomiting, pains of the limbs, knees, and back, a ſenſe of cold, ſometimes continuing for hours, but ſeldom amounting to the degree called horror: the pulſe, for the moſt part is ſmall, weak, and eaſily compreſſed, ſometimes, but not always, frequent, irregular, heſitating, and tremulous or creeping; ſometimes in appearance tenſe, or confined in volume, with an impreſſion of obſtruction; the animal heat, on the ſurface of the body, is ſeldom greater than natural—the internal ſenſation far exceeds; the ſkin is uſually dry; if damp, it is unpleaſant to the touch; the feelings are uncomfortable; and the appearance of the eye and countenance indicates a deſponding mind;—the countenance is uſually dirty and lurid, ſallow, like a ſickly plant, or fading leaf: the eye ſad, but ſeldom inflamed; it is inanimate, and ſometimes gloſſy: the tongue is ſometimes covered with a white mucous coat, ſometimes ſo thin that the red colour ſhines through it, ſometimes of conſiderable thickneſs; it is ſeldom brown, and it is oftener moiſt than dry; ſometimes it is pale, and ſmooth, and clean, the mouth abounding with a ropy ſaliva: the ſeat of the pain of the head is generally over the eyes, almoſt always in the forehead or temples, in many caſes ſo oppreſſive, as to occaſion ſtupor like intoxication; ſtrangury, or want of power over the urinary diſcharge, is not uncommon.

" The head-ach, for the moſt part, abates, or ſuffers change of form, at the expiration of the firſt twelve hours; and where tumult and irritation had been conſpicuous in the beginning, they generally diminiſh about this time, and partial ſweat, with temporary relief, enſues; yet this abatement is neither conſtant, nor of long duration, the ſufferings and diſtreſſes recur in a few hours, particularly the pain of the head, the unnatural appearances of the eye increaſe, the countenance becomes more dingy and greaſy, or flaccid, dry, and withered; the pulſe is ſometimes more frequent than natural, generally ſmall, and confined, yet in many inſtances it does not perceptibly differ from the pulſe of health, unleſs in want of energy and expanſion; the thirſt is ſeldom great, but the lips are uſually dry, the mouth clammy, and the taſte depraved; the tongue is for the moſt part covered with a white mucous paſte, generally moiſt; the ſenſations at ſtomach are unpleaſant; nauſea, anxiety, ſighing, and deep breathing, are uſual. The ſtate of the ſkin, in this form of diſeaſe, deſerves remark,—it is dry, withered, and thickens by a rapid progreſs, becoming impervious, or in a manner cut off from the free current of circulation: the face face of things proceeds then very much in one tenor; a torpor, or impaired ſenſibility, poſſeſſing the functions, the progreſs to deſtruction is ſilent, and often unperceived."

III. In the *third* form, the prominent feature is, an aſpect of countenance dark and cloudy, as in ſcurvy.

"This form of diſeaſe appeared principally among thoſe who were removed from a more pure and cool, to a confined and immoderately hot air, whoſe habits were full, and who were reſtrained from the active uſe of their limbs. In this manner it was common under the removal of troops from poſt to poſt, or under the indulgences and reſt, which uſually followed military excurſions in St. Domingo.

"The formal attack is ſometimes preceded by heavineſs and oppreſſion, ſometimes the attack, from a ſtate of high health, is ſudden and inſtantaneous, the ſymptoms are common to the claſs of fevers. Head-ach is among the firſt; it is intenſely ſevere, or heavy and oppreſſive, it is accompanied by giddineſs, faintneſs, often by an undefinable uneaſineſs; a ſenſe of cold is often of long continuance, but ſeldom amounts to horror; the pulſe is uſually frequent, ſmall, oppreſſed, and weak, or without energy of ſtroke; increaſe of heat is uncertain, and, where it is perceived, it is ordinarily of ſhort duration; the ſkin is dry—if moiſt, it is damp, unpleaſant, and greaſy—it is ſometimes preternaturally cool; the countenance is livid, and of a duſky hue, dark, and overcaſt, as in ſcurvy; the eye is uncommonly clear and gloſſy, of a pearly white, and vacant expreſſion; the thirſt is irregular, ſeldom much increaſed; ſighing and deep breathing are frequent; a ſenſe of ſtricture, or inability of expanding the cheſt, without pain, is common; the general feelings are unſatisfactory, and pains of the loins and limbs are ſometimes ſevere: ſoreneſs of the fleſh is a frequent complaint; nauſea is not unuſual, and even vomitting ſometimes takes place, but ſeldom in a material degree; the tongue is rarely ſuch as is called foul, but it is often covered with tough ſaliva, and ſometimes with a whitiſh paſte; it is ſeldom dry, ſometimes it is clean, ſmooth, and without the prominence of papillæ."

IV. The *fourth* form, which includes fevers of type.

"The cauſe of endemic fever, continued, remitting, or intermitting, is one; but great variety is produced in the form, and manner of action. The diſeaſe, in the more violent forms is, or appears to be, continued in ſome ſituations, in others it is remitting, and of regular type. In wet weather, and on ſwampy grounds, the endemic of the country is uſually remitting in form; and under this form exhibits appearances of jaundiced yellowneſs, of black vomiting, purgings of black matter, hæmorrhage from different parts of of the body, petechiæ, lividneſs, &c. The tertian, or the compounds of the tertian, chiefly prevail; but, in ſeveral inſtances, a paroxyſm ſeems to continue for forty-eight hours without remiſſion; the third day is quiet, but fever re-appears on the fourth, from which period the type is ſometimes regular and diſtinct, with paroxiſms on the alternate days—ſometimes the powers of life are ſuffocated by the acceſſion; hence the fourth, or the morning of the fifth, is often a fatal day in the fevers of ſtrangers, whether continued or remitting. The ſymptoms of the paroxiſms are of the ſame form and kind as where the form is continued, or without remiſſion; they ſubſide at a certain period, and at a certain period re-commence, going on for a limitted ſpace of time in this alternate action, and ceſſation from action. The circumſtances connected with theſe alternate ſtates of morbid action, and ceſſation from action, throw a great deal of light on the general operation of febrile cauſes.

"Correſponding with the firſt form of continued fever, the ſymptoms of the paroxiſms of the remittent are violent, with great irritation, and ſtrong action of the vaſcular ſyſtem, an ardent, pungent, and ſometimes an exceſſively cauſtic heat, ſevere local pains, anxiety, reſtleſſneſs, anguiſh at ſtomach, nauſea, ſickneſs, vomiting, hurried reſpiration, ſevere and diſtreſſing head-ach, pain of the eyes, delirium, &c. Theſe ſufferings abate at a certain period, but they do not often terminate by copious perſpiration; they recur, ſubſide, and recur again at intervals, till a critical period arrives—frequently the ſeventh day, when they ceaſe finally, or ſigns appear of a fatal termination. During this courſe, ſymptoms often ariſe, ſimilar to the ſymptoms of the concentrated continued fevers; the ſkin becomes dry, dingy, and withered, jaundiced yellowneſs makes its appearance in the eyes, black vomiting ſometimes takes place, and in many inſtances hæmorrhage, or exudations of blood from different parts of the body—moſt frequently the tract of alimentary canal.

"A mode of action is alſo diſcovered under remitting form, in ſome manner correſponding with the ſecond form of continued fever. The pulſe is frequent, ſmall and low, eaſily compreſſed; without energy and force; the ſkin cool, damp and greaſy, or dry, withered and duſky, the head-ach frequently ſevere, the countenance inanimate and depreſſed, reſtleſſneſs is conſiderable, with anxiety at ſtomach and uncomfortable ſenſations; ſometimes the alimentary canal is principally affected; there is thirſt, a dry tongue, vomiting or purging returning at periods, but in no extraordinary degree of violence; upon the whole, the indiſpoſition ſeems ſlight, the patient walks about, neither well, nor to common apprehenſion, materially ill. At a certain period, frequently on the fifth, ſometimes on the ſeventh day, the nature of the action changes or becomes more intenſe in degree; the head head is affected with coma, ſometimes with a muttering delirium, the pulſe becomes weak and ſunk, the action of the fibre is impaired, in ſome manner ſuſpended; a ſpecies of paralyſis takes place, the heat ſinks below natural. This mode of action continues twelve, ſometimes twenty-four hours; the powers gradually emerge, and are again oppreſſed by a ſimilar ſuſpenſion. Sometimes the animal heat is, in a manner, extinguiſhed, the pulſe totally ſuppreſſed; a mode of action which ceaſes and returns at intervals, and is known to be leſs fatal than threatening, by certain ſenſations of eaſe and quiet,—the ſerene and cheerful eye and countenance which accompany it.

"The remitting alſo appears, in many inſtances,under a mode of action correſponding with the third form of continued fever. A livid dingineſs overſpreads the countenance from the commencement of the diſeaſe, a general torpor marks the exiſtence of the paroxyſm; the blood ſeems to ſtagnate in the extreme veſſels, particularly under the nails, where a blackneſs, like a blemiſh, grows out in recovery; important organs, the lungs or brain, are often oppreſſed during this action of the febrile cauſe, and death is the conſequence; when this is not the caſe, the powers of life emerge, till the renewal of the action produces a ſimilar ſuſpenſion, and frequently a fatal termination. This is the ordinary courſe in the more intenſe degrees, under the operation of powerful cauſes; in more common circumſtances, the commencement is ſlight, the type is regular, and remiſſions, though not terminated by copious perſpirations or other evacuations, upon the whole diſtinct: about the fifth or ſeventh day, a change of action takes place; the powers of life are in a manner ſuſpended, or a great degree of torpor, in all the animal actions, ſupervenes; the circulation is heavy and oppreſſed, the countenance duſky and grim, dark, like mahogany, often greaſy, damp, and dirty; general torpor and diminution of all the ſecretions mark the period of the paroxyſm; theſe oppreſſions vaniſh, in ſome degree, after twelve or twenty-four hours, and again return at a given period.—Such a form of diſeaſe is frequent in the more unhealthy ſituations of St. Domingo, in the ſummer and autumnal months: it occurs occaſionally in the more healthy, in the months of October and November."

Chap. VI. Contains ſome appearances obſerved on diſſection.

In the next chapters, Dr. Jackſon compares the characteriſtics of endemic and contagious fever, and lays down the prognoſis in each; he makes a few obſervations on critical days and proximate cauſes.

Chap. XI. *On the* Cure *of Fever.*

This part of the ſubject does not appear to be ſo much indebted to our Author as ſeveral of the preceding. A talent for obſervation and deſcription, joined to suitable opportunities, may always improve the science of medicine, by furnishing the requisite data. Dr. Jackson has availed himself of these; but on the method of cure, he says, "Medical science has i general advanced; some parts of it have made considerable progress, but the cure of fever appears to be stationary, if not retrograde. Books have been written upon the subject without number; infallible methods fill the pages of authors, and important discoveries are communicated in every new publication; yet, men die, as in the days of ignorance. The cure of fever, it must be acknowledged, is difficult, and capable of little perfection, in the state of progress at which the disease is usually submitted to the care of physicians. But though difficult, it might be presumed, that something could not well fail to be discovered, from the unwearied research of writers. The subject has filled volumes, yet it does not appear that a general principle is attained: the result, consequently, is a mass of contradictions—a collection of opinions, not always candidly and ingenuously represented. The author of this outline has felt the inconvenience, and now ventures to suggest some hints, which he hopes may in time lead to a remedy:—the laws of health are uniform and regular—even disease obeys a rule. If the precise form of diseased action could be ascertained, the method of cure might be laid down upon a sure foundation: this unfortunately is not the case; but even the knowledge of a principle, by which this action may be inverted, is of value. It implies, it must be confessed, an experiment apparently at random, but, under certain conditions, an experiment of safety.

"According to the manner in which the author has long viewed this subject, the plan of cure divides itself into two parts; viz. into the cure of a fever forming—and into the cure of a fever formed. In the first, art is capable of doing every thing; and it consequently ought not to leave any thing to nature:—in the second, a form of things has taken place, or a chain of operation is established, which can seldom be broken forcibly, consistent with safety to life; art can do little, and the little which can be done, requires caution and judgment; for, to act and not to do harm, under such a condition of things, is not ordinarily a matter of indifference.

"The cause of fever, whatever it may be, or whatever may be the direct mode of its operation, visibly and indisputably changes natural and healthy action, generally or locally, into action diseased and unnatural.—To invert this operation, to originate a new train of motions, analogous to those of health, is the fundamental principle of cure: and this much is certain, that if the object be undertaken at the proper period, the plan judiciously laid, and followed up with vigour, the end seldom fails of being attained. Decided practices, of whatever description, succeed; and the complete and perfect recovery of health is often the effect of directly opposite means; means; on the contrary, if the early period of diſeaſe be paſt, ſo that the organization of parts is injured, or deeply impreſſed with a figure of unnatural action, the conduct of the cure is a matter of great nicety, and requires great caution; the indications fluctuate and vary according to circumſtances; ſymptoms, or modes of action, which threaten danger to life, will then be watched and warded off; but termination, or decided cure, muſt be left to the periods of change; for though criſis, by judicious exertion ſometimes actually is, and often is capable of being, rendered more complete than it otherwiſe would be, it is doubtful to what extent it can be accelerated.—Theſe periods of change are important to the phyſician; and in fever, completely formed or advanced in courſe, muſt principally regulate his conduct."

Dr. Jackſon firſt treats of the cure of *contagious fever*; and in Sec. II. p. 263, of the cure of endemic fever. The principal novelty in the treatment of both, conſiſts in attempts to cut ſhort the progreſs of morbid action, or diſſever the half-formed morbid aſſociations in the commencement of the diſeaſe.

This is attempted, according to the nature of the fever, and the ſtate of the patient, by V.S. emetics, calomel, bliſters, fomentations, and above all, by ſuddenly contraſting the hot and cold bath.

After the fever is completely formed, the cure recommended by Dr. Jackſon does not materially differ from that of other authors.

The extracts we have given from the deſcriptive part, are doubtleſs ſufficient to convey an idea of the originality, accuracy, and depth of reſearch of our author: we truſt they are ſufficient to induce our readers to conſult the work itſelf.

